# Sex differences in body composition in people with prediabetes and type 2 diabetes as compared with people with normal glucose metabolism: the Maastricht Study

**DOI:** 10.1007/s00125-023-05880-0

**Published:** 2023-02-20

**Authors:** Rianneke de Ritter, Simone J. S. Sep, Marleen M. J. van Greevenbroek, Yvo H. A. M. Kusters, Rimke C. Vos, Michiel L. Bots, M. Eline Kooi, Pieter C. Dagnelie, Simone J. P. M. Eussen, Miranda T. Schram, Annemarie Koster, Martijn C. G. Brouwers, Niels M. R. van der Sangen, Sanne A. E. Peters, Carla J. H. van der Kallen, Coen D. A. Stehouwer

**Affiliations:** 1 Department of Internal Medicine, Maastricht University Medical Center+, Maastricht, the Netherlands; 2 CARIM School for Cardiovascular Diseases, Maastricht University, Maastricht, the Netherlands; 3 Adelante, Center of Expertise in Rehabilitation and Audiology, Hoensbroek, the Netherlands; 4 Julius Center for Health Sciences and Primary Care, University Medical Center Utrecht, Utrecht University, Utrecht, the Netherlands; 5 Leiden University Medical Center, Department of Public Health and Primary Care/LUMC-Campus, The Hague, the Netherlands; 6 Department of Radiology and Nuclear Medicine, Maastricht University Medical Center+, Maastricht, the Netherlands; 7 Department of Epidemiology, Maastricht University, Maastricht, the Netherlands; 8 CAPHRI Care and Public Health Research Institute, Maastricht University, Maastricht, the Netherlands; 9 Heart and Vascular Center, Maastricht University Medical Center+, Maastricht, the Netherlands; 10 MHeNs School for Mental Health and Neuroscience, Maastricht University Medical Center+, Maastricht, the Netherlands; 11 Department of Social Medicine, Maastricht University, Maastricht, the Netherlands; 12 Department of Cardiology, Amsterdam UMC, University of Amsterdam, Amsterdam, the Netherlands; 13 The George Institute for Global Health, Imperial College London, London, UK

**Keywords:** Body composition, DEXA, Fat mass, Lean mass, Liver fat, MRI, Prediabetes, Sex differences, Type 2 diabetes, Women

## Abstract

**Aims/hypothesis:**

Obesity is a major risk factor for type 2 diabetes. However, body composition differs between women and men. In this study we investigate the association between diabetes status and body composition and whether this association is moderated by sex.

**Methods:**

In a population-based cohort study (*n*=7639; age 40–75 years, 50% women, 25% type 2 diabetes), we estimated the sex-specific associations, and differences therein, of prediabetes (i.e. impaired fasting glucose and/or impaired glucose tolerance) and type 2 diabetes (reference: normal glucose metabolism [NGM]) with dual-energy x-ray absorptiometry (DEXA)- and MRI-derived measures of body composition and with hip circumference. Sex differences were analysed using adjusted regression models with interaction terms of sex-by-diabetes status.

**Results:**

Compared with their NGM counterparts, both women and men with prediabetes and type 2 diabetes had more fat and lean mass and a greater hip circumference. The differences in subcutaneous adipose tissue, hip circumference and total and peripheral lean mass between type 2 diabetes and NGM were greater in women than men (women minus men [W–M] mean difference [95% CI]: 15.0 cm^2^ [1.5, 28.5], 3.2 cm [2.2, 4.1], 690 g [8, 1372] and 443 g [142, 744], respectively). The difference in visceral adipose tissue between type 2 diabetes and NGM was greater in men than women (W–M mean difference [95% CI]: −14.8 cm^2^ [−26.4, −3.1]). There was no sex difference in the percentage of liver fat between type 2 diabetes and NGM. The differences in measures of body composition between prediabetes and NGM were generally in the same direction, but were not significantly different between women and men.

**Conclusions/interpretation:**

This study indicates that there are sex differences in body composition associated with type 2 diabetes. The pathophysiological significance of these sex-associated differences requires further study.

**Graphical abstract:**

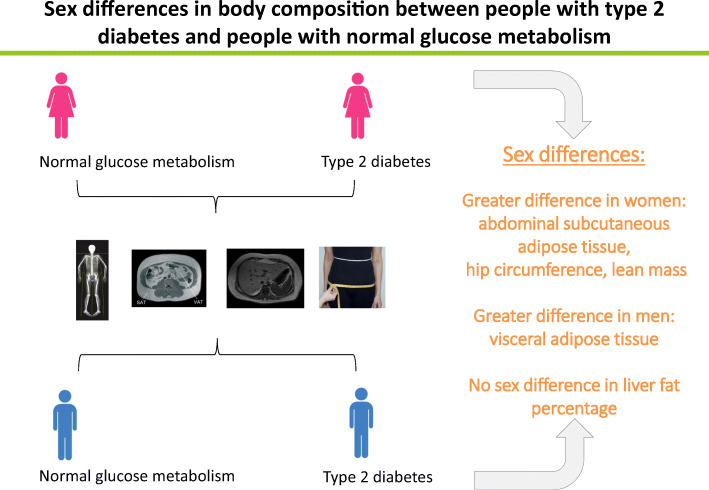

**Supplementary Information:**

The online version contains peer-reviewed but unedited supplementary material available at 10.1007/s00125-023-05880-0.

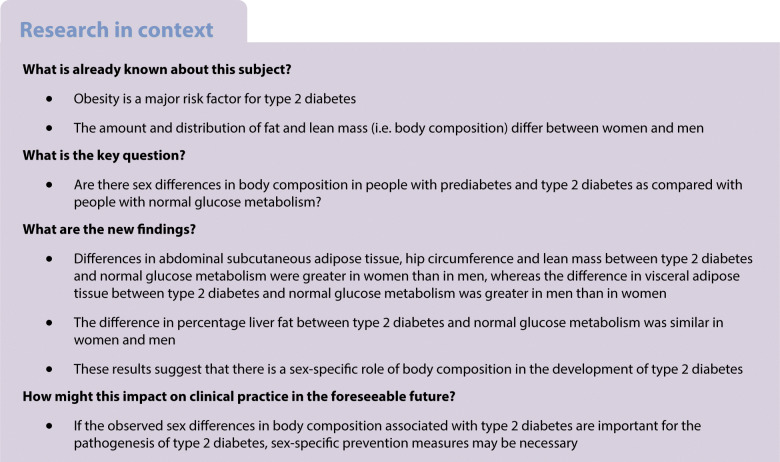



## Introduction

Obesity is associated with a proinflammatory state and dyslipidaemia and is a major risk factor for type 2 diabetes [1]. The amount and distribution of fat and lean mass (i.e. body composition) differ between women and men, with women having proportionally more fat mass and men more muscle mass [2].

Sex differences have been reported in the association of excess body fat with type 2 diabetes [3]. In general, women have a higher BMI at diagnosis of type 2 diabetes [3]. Women generally have a greater amount of total body fat than men, but an increase in body fat appears to have a smaller effect on their insulin sensitivity [4]. The transition from normoglycaemia to type 2 diabetes may be associated with more fat accumulation in women, because they tend to store excess fat first in less metabolically harmful regions (i.e. subcutaneously and on their lower extremities) and subsequently in more harmful regions (e.g. as visceral adipose tissue [VAT] in the abdominal region) [4, 5]. In contrast, men predominately store fat more rapidly as VAT, which is associated with metabolic disturbances and higher risks of type 2 diabetes and CVD [4].

Meanwhile, the role of lean mass is ambiguous. One study reported that a higher lean mass was significantly associated with a lower risk of diabetes in women, and this association was directionally similar in men [6]. Additionally, in both sexes, hyperglycaemia has been associated with a lower lean mass [7, 8]. While lean mass may be beneficial for glucose metabolism, it has also been suggested that greater lean mass may not protect against insulin resistance [9, 10]. More specifically, among men, greater lean mass accompanied by greater fat mass may be detrimental for glucose regulation, whereas, among women, greater fat mass is the major determinant of glucose intolerance [9]. Additionally, people with both a high fat and a high lean mass were shown to have the most unfavourable cardiometabolic risk profile [10]. Therefore, it is important to take both fat and lean mass into account with regard to diabetes development. However, no large studies are available that have analysed sex differences in body composition associated with prediabetes (i.e. impaired fasting glucose and/or impaired glucose tolerance) and/or type 2 diabetes.

In view of these considerations, more insight into sex differences in the amount of fat and lean mass between people with (pre)diabetes and people with normal glucose metabolism (NGM) could contribute to a greater understanding of the sex-specific role of body composition in the development of type 2 diabetes. Therefore, we investigated sex-specific associations, and differences therein (i.e. interactions), of (pre)diabetes with dual-energy x-ray absorptiometry (DEXA)- and MRI-derived measures of body composition and with hip circumference.

## Methods

### Study design and population

Data from the Maastricht Study, an observational prospective population-based cohort study, were used in this study. The rationale and methodology have been described previously [11]. In brief, the study focuses on the aetiology, pathophysiology, complications and comorbidities of type 2 diabetes and is characterised by an extensive phenotyping approach. All individuals aged between 40 and 75 years and living in the southern part of the Netherlands were eligible for participation. Participants were recruited through mass media campaigns and from the municipal registries and the regional Diabetes Patient Registry through mail-outs. Recruitment was stratified according to known diabetes status, with an oversampling of individuals with type 2 diabetes, for reasons of efficiency. This study includes cross-sectional data from the first 7689 participants, who completed the baseline survey between November 2010 and December 2017. All examinations of participants were performed within a time frame of 3 months, except for the DEXA and MRI scans. DEXA measurements were implemented from January 2015 onwards, with a mean lag time of 2.6 years. MRI measurements were implemented from December 2013 onwards and had a mean lag time of 1.2 years. The study was approved by the institutional medical ethics committee (NL31329.068.10) and the Minister of Health, Welfare and Sport of the Netherlands (permit 131088-105234-PG). All participants gave written informed consent. For the current study, individuals with other types of diabetes than type 2 diabetes were excluded (*n*=50).

### Assessment of body composition

**Fig. 1:**
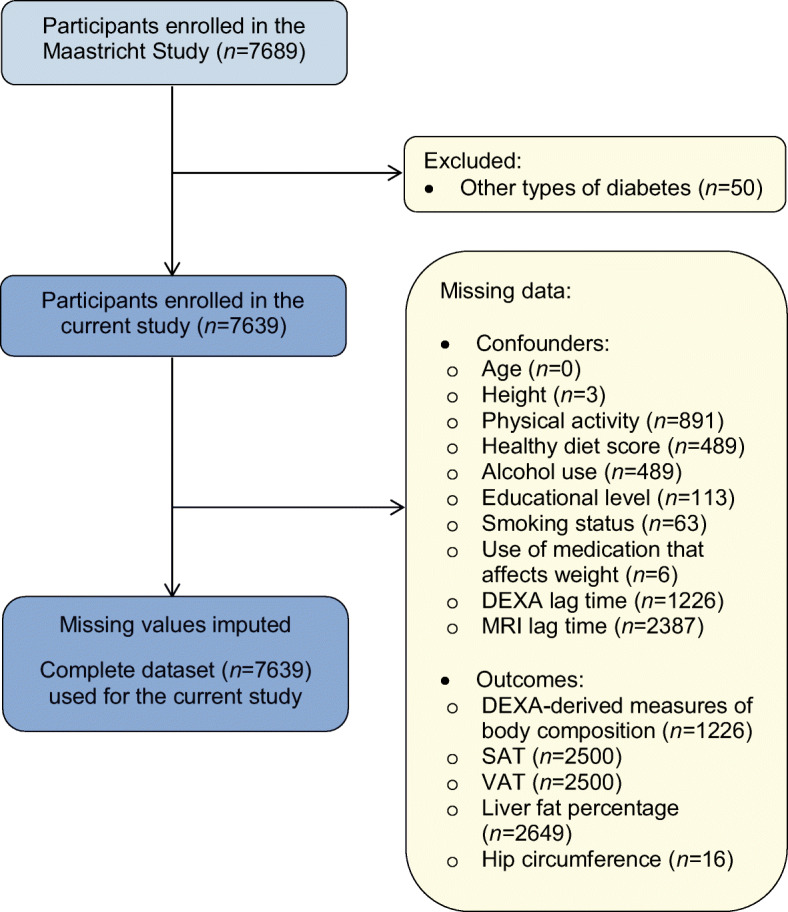
Flowchart of study participants

A DEXA scanner was used to assess participants’ fat and lean mass (electronic supplementary material [ESM] Fig. 1) as described in ESM Methods. MRI was performed to determine the amount of abdominal subcutaneous adipose tissue (SAT) and VAT and liver fat percentage (ESM Fig. 1, ESM Methods).

Because of a technical error in the case of 250 participants, measurements of the amount of SAT (*n*=250) and VAT (*n*=28) were incomplete. We estimated these values as described in ESM Methods.

We used hip circumference, determined as described elsewhere [11], as a proxy for thigh and buttock fat [12] (ESM Fig. 1).

### Assessment of glucose metabolism status

To determine glucose metabolism status (GMS), all participants underwent a standardised 2 h 75 g OGTT after fasting overnight. Further details on the assessment of GMS, as well as the assessments of covariates and population characteristics, are described in ESM Methods.

### Statistical analyses

SSPS version 27.0 (IBM, USA) was used for the statistical analyses. Population characteristics were described as mean ± SD and median (IQR), for normally and non-normally distributed variables, respectively, or *n* (%) for discrete variables. Variables were log-transformed if residuals were skewed.

Sex and the interaction of sex-by-(pre)diabetes need to be distinguished as potential determinants, as described in more detail elsewhere [13]. We used generalised linear models to estimate adjusted (model 3 as described below) sex-specific amounts of DEXA-estimated fat and lean mass, MRI-estimated amounts of VAT and SAT and liver fat percentage, and hip circumference in participants with NGM, prediabetes and type 2 diabetes. We used linear regression analyses to test whether sex was a determinant in these associations. Our main goal was to investigate sex-by-(pre)diabetes interactions; therefore, we used linear regression analyses (based on two-sided tests) to estimate sex-specific associations, and differences therein (i.e. interactions), of prediabetes and type 2 diabetes (reference category: NGM) with DEXA-estimated fat and lean mass, with MRI-estimated VAT, SAT and liver fat percentage and with hip circumference. To test for sex differences, interaction terms of sex-by-dummy-coded (pre)diabetes status (i.e. sex-by-prediabetes and sex-by-type 2 diabetes) were incorporated into the models. Several sets of adjustments were made. Model 1 was adjusted for age and height. We adjusted for height as a measure of body size in the associations with fat and lean mass, expressed as an amount (g) or area (cm^2^). Thus, only liver fat percentage was not adjusted for height, as we considered height not to be a potential confounder. Model 2 was additionally adjusted for other potential confounders, that is, physical activity, healthy diet score, educational level, alcohol consumption and smoking status. If total or peripheral lean mass was the outcome, model 2 was additionally adjusted for total fat mass. Model 3 (main model) was additionally adjusted for the use of medication that may cause weight gain and/or weight loss as a side effect. For each potential confounder included, an interaction term (sex-by-potential confounder) was also incorporated in the same models to ensure that the adjustments made in the interaction models would vary by sex as they do in the sex-specific models [14]. For the interactions of sex with (pre)diabetes, *p*<0.05 was considered statistically significant and the results are presented with 95% CIs. Multiple imputation was performed for both potential confounders and outcomes (i.e. measures of body composition). The percentage of missing values was a maximum of 11.7% for potential confounders and 34.7% for outcomes (Table 1, Fig. 1). We imputed data using multiple imputation by chained equations under the assumption that data were missing at random. We used predictive mean matching to impute 20 datasets with ten iterations for each dataset. For the main analysis, we pooled the results across all imputed datasets with the use of Rubin’s rule [15].

**Table 1:** Study population characteristics according to sex and GMS

Characteristic	Women (*N*=3788)			Men (*N*=3851)		
	NGM (*n*=2632)	Prediabetes (*n*=525)	T2D (*n*=631)	NGM (*n*=1973)	Prediabetes (*n*=616)	T2D (*n*=1262)
Demographic characteristics and health measures						
Age (years)	57.4 ± 8.5	61.6 ± 8.5	62.3 ± 8.2	58.7 ± 8.7	62.8 ± 7.9	63.4 ± 7.6
Height (cm)^a^	165 ± 6	163 ± 6	163 ± 6	178 ± 7	177 ± 6	176 ± 7
Education level (*n*)^a^						
Low	842 (32.4)	229 (44.6)	364 (59.1)	463 (23.6)	200 (32.9)	509 (41.3)
Medium	751 (28.9)	136 (26.5)	147 (23.9)	532 (27.2)	166 (27.3)	346 (28.1)
High	1005 (38.7)	149 (29.0)	105 (17.0)	964 (49.2)	241 (39.7)	377 (30.6)
Smoking status (*n*)^a^						
Never	1105 (42.3)	195 (37.4)	248 (39.9)	781 (39.7)	183 (29.9)	309 (24.9)
Former	1203 (46.0)	265 (50.9)	278 (44.8)	909 (46.2)	351 (57.4)	733 (59.1)
Current	307 (11.7)	61 (11.7)	95 (15.3)	276 (14.0)	78 (12.7)	199 (16.0)
Alcohol use (g/day)^b^	8.5 ± 9.3	8.3 ± 10.4	5.1 ± 8.2	16.3 ± 14.7	18.7 ± 19.4	13.8 ± 16.0
Healthy diet score (score)^b^	81 ± 13	79 ± 13	77 ± 14	74 ± 15	73 ± 14	70 ± 14
Systolic blood pressure (mmHg)	126 ± 17	134 ± 18	139 ± 18	134 ± 15	140 ± 17	143 ± 18
Diastolic blood pressure (mmHg)	72 ± 9	75 ± 10	76 ± 10	77 ± 9	79 ± 10	78 ± 10
Use of blood pressure-lowering medication (*n*)^a^	520 (19.8)	222 (42.4)	437 (69.3)	516 (26.2)	304 (49.4)	909 (72.1)
Total cholesterol (mmol/l)	5.6 ± 1.0	5.6 ± 1.1	4.8 ± 1.0	5.3 ± 1.0	5.2 ± 1.1	4.3 ± 1.0
HDL-cholesterol (mmol/l)	1.8 ± 0.5	1.6 ± 0.4	1.5 ± 0.4	1.4 ± 0.4	1.3 ± 0.4	1.2 ± 0.3
LDL-cholesterol (mmol/l)	3.3 ± 0.9	3.3 ± 1.0	2.6 ± 0.9	3.3 ± 0.9	3.1 ± 1.0	2.3 ± 0.9
Triglycerides (mmol/l)	1.1 ± 0.6	1.6 ± 0.9	1.7 ± 0.9	1.3 ± 0.7	1.7 ± 1.2	1.8 ± 1.1
Use of lipid-modifying medication (*n*)^a^	321 (12.2)	151 (28.8)	425 (67.4)	394 (20.0)	240 (39.0)	938 (74.4)
Use of medication that affects weight (*n*)^a,c^	421 (16.0)	142 (27.1)	285 (45.2)	284 (14.4)	178 (28.9)	526 (41.7)
Moderate/vigorous physical activity (h/week)^b^	6.1 ± 4.5	5.0 ± 3.8	4.5 ± 4.2	5.9 ± 4.5	5.1 ± 4.5	4.2 ± 4.2
Postmenopausal status (*n*)^a^	1941 (74.9)	432 (83.9)	537 (87.9)	n/a	n/a	n/a
Use of postmenopausal hormone replacement medication^a^	55 (2.8)	10 (2.3)	14 (2.6)	n/a	n/a	n/a
Measures of body composition						
Total body fat mass (kg)^b^	27.1 ± 7.9	30.7 ± 8.7	34.3 ± 10.2	23.4 ± 6.7	26.9 ± 7.5	29.2 ± 8.6
Total body lean mass (kg)^b^	41.4 ± 5.1	42.6 ± 5.8	44.9 ± 6.5	58.3 ± 6.5	59.6 ± 7.2	60.1 ± 7.5
Peripheral fat mass (kg)^b^	13.5 ± 3.9	14.3 ± 4.4	15.2 ± 4.8	9.8 ± 2.8	10.8 ± 3.3	11.3 ± 3.5
Peripheral lean mass (kg)^b^	17.4 ± 2.5	17.7 ± 2.7	18.4 ± 3.1	25.9 ± 3.2	26.1 ± 3.5	25.8 ± 3.6
Trunk adipose tissue (kg)^b^	12.6 ± 4.5	15.4 ± 4.8	18.1 ± 6.0	12.4 ± 4.1	14.9 ± 4.5	16.7 ± 5.3
Gynoid fat mass (kg)^b^	3.5 ± 1.0	3.9 ± 1.1	4.1 ± 1.2	4.8 ± 1.3	5.0 ± 1.4	5.2 ± 1.6
SAT (cm^2^)^b^	220.9 ± 92.1	256.0 ± 99.7	283.7 ± 107.8	184.7 ± 68.7	206.1 ± 72.8	214.8 ± 79.4
VAT (cm^2^)^b^	99.0 ± 56.1	143.1 ± 64.4	181.4 ± 78.8	176.8 ± 88.0	229.7 ± 88.5	279.2 ± 107.9
Liver fat percentage (%)^b^	2.4 (1.6–3.9)	4.2 (2.4–8.4)	6.3 (3.2–11.9)	3.0 (2.1–5.0)	4.7 (2.9–8.1)	6.4 (3.6–11.5)
BMI (kg/m^2^)	25.2 ± 4.0	27.7 ± 4.7	30.7 ± 5.6	26.1 ± 3.2	28.0 ± 3.7	29.3 ± 4.6
Waist circumference (cm)	86 ± 11	93 ± 12	102 ± 14	96 ± 10	103 ± 10	108 ± 12
Hip circumference (cm)	101 ± 9	104 ± 10	109 ± 12	99 ± 6	102 ± 7	104 ± 8
Measures of glucose metabolism						
Fasting glucose (mmol/l)	5.0 ± 0.4	5.7 ± 0.6	7.5 ± 1.8	5.2 ± 0.4	6.0 ± 0.5	8.0 ± 2.0
Post-load glucose (mmol/l)^d^	5.4 ± 1.1	8.6 ± 1.2	14.5 ± 3.9	5.3 ± 1.2	8.0 ± 1.8	14.2 ± 3.7
HbA_1c_ (mmol/mol)	35.3 ± 3.8	38.0 ± 4.4	50.2 ± 11.2	35.3 ± 3.9	38.1 ± 4.5	51.6 ± 11.7
HbA_1c_ (%)	5.4 ± 0.4	5.6 ± 0.4	6.7 ± 1.0	5.4 ± 0.4	5.6 ± 0.4	6.9 ± 1.1
Use of oral drugs for T2D (*n*)	n/a	n/a	409 (64.8)	n/a	n/a	897 (71.1)
Use of insulin for T2D (*n*)	n/a	n/a	100 (15.8)	n/a	n/a	259 (20.5)

Data are presented as mean ± SD, median (25th–75th percentile) in case of a skewed distribution or *n* (%)

^a^Missing data <5% per variable: height, *N=*7636; education level, *N*=7526; smoking status, *N*=7576; blood pressure-lowering medication, *N*=7633; lipid-modifying medication, *N*=7633; medication that affects weight, *N*=7633; postmenopausal status (women only), *N*=3719; hormone replacement medication (postmenopausal women only), *N*=2910

^b^Missing data >5% per variable: alcohol use*, N*=7150; healthy diet score, *N*=7150; moderate/vigorous physical activity, *N*=6748; DEXA-derived measures of body composition, *N*=6413; SAT and VAT, *N*=5139; liver fat percentage, *N*=4990

^c^Use of medication that affects weight was defined as using one or more of the following medications: hormonal contraceptives, antidepressants, antipsychotic drugs, lithium, medicinal cannabis, β-blockers, anti-epileptics (i.e. valproic acid, gabapentin, carbamazepine and topiramate), mineralocorticoids (i.e. fludrocortisone), glucocorticoids (i.e. betamethasone, dexamethasone, methylprednisolone, prednisolone, prednisone, triamcinolone [acetonide], hydrocortisone and cortisone)

^d^Missing data for 23% of individuals with type 2 diabetes per protocol

n/a, not applicable; T2D, type 2 diabetes

From an aetiological perspective, we were interested in the potential effect of body composition on the development of (pre)diabetes in men and women. Although it may seem counterintuitive, we specifically chose to analyse the data with (pre)diabetes as the determinant and measures of body composition as outcomes, and not the other way around, because results of analyses with body composition measures as determinants are difficult to interpret. For example, men have more VAT than women and therefore a 1 cm^2^ increase in VAT is a relatively smaller increase for men than for women. Hence, the results, for example the odds of having (pre)diabetes compared with NGM per 1 cm^2^ increase in VAT, are difficult to compare between women and men. Similarly, men also have a higher SD of VAT than women, because of their greater amount of VAT, so comparing SDs between men and women would also give results that are difficult to interpret.

In the current analyses, the results are expressed as linear regression coefficients, which represent mean differences (βs) or geometric mean ratios (GMRs) for measures of body composition (g or cm^2^) according to (pre)diabetes status (reference category: NGM). The results are presented for men and women separately and can be seen as a snapshot indicating the amount of fat and lean mass in (pre)diabetes compared with NGM.

To investigate the robustness of the results obtained by the above analyses we performed several sensitivity analyses as described in ESM Methods.

## Results

The study population consisted of 3788 women (age 58.8 ± 8.7 years) and 3851 men (age 60.9 ± 8.5 years). Of these individuals, 4605 (57.2% women) had NGM, 1141 (46.0% women) had prediabetes and 1893 (33.3% women) had type 2 diabetes (Table 1).

### DEXA-derived measures of body composition

#### Sex as determinant

**Fig. 2:**
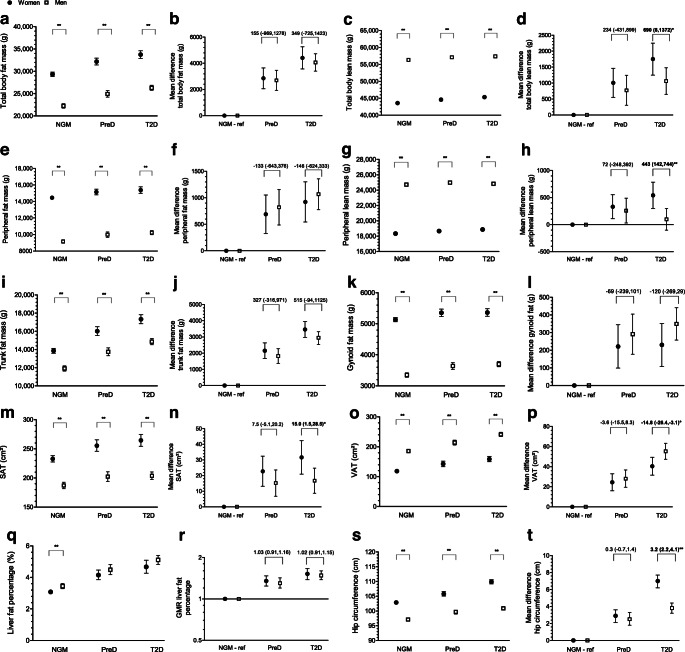
(**a**, **c**, **e**, **g**, **i**, **k**, **m**, **o**, **q**, **s**) Sex as a determinant of body composition in participants with NGM, prediabetes and type 2 diabetes: total body fat mass (**a**), total body lean mass (**c**), peripheral fat mass (**e**), peripheral lean mass (**g**), trunk fat mass (**i**), gynoid fat mass (**k**), SAT (**m**), VAT (**o**), liver fat percentage (**q**) and hip circumference (**s**). The graphs shows adjusted (fully adjusted model) sex-specific means and corresponding 95% CIs. Statistically significant adjusted (fully adjusted model) differences in body composition between women and men (sex differences) are indicated. *p<0.05, **p<0.01. (**b**, **d**, **f**, **h**, **j**, **l**, **n**, **p**, **r**, **t**) Sex-by-(pre)diabetes as a determinant of body composition in participants with NGM, prediabetes and type 2 diabetes: total body fat mass (**b**), total body lean mass (**d**), peripheral fat mass (**f**), peripheral lean mass (**h**), trunk fat mass (**j**), gynoid fat mass (**l**), SAT (**n**), VAT (**p**), liver fat percentage (**r**) and hip circumference (**t**). The graphs show adjusted (fully adjusted model) sex-specific mean differences (for all body composition variables except liver fat percentage) or GMRs (for liver fat percentage; **r**) between (pre)diabetes and NGM (reference category). Results are expressed as adjusted (fully adjusted model) linear regression coefficients and corresponding 95% CIs. Statistically significant differences between women and men (sex differences) are indicated. *p<0.05, **p<0.01. preD, prediabetes; ref, reference; T2D, type 2 diabetes

Compared with men, independent of GMS, women had significantly higher levels of total fat, peripheral fat, trunk fat and gynoid fat mass (Fig. 2a,e,i,k). In contrast, men, independent of GMS, had significantly higher levels of total and peripheral lean mass than women (Fig. 2c,g).

#### Sex-by-(pre)diabetes interaction

Compared with their NGM counterparts, both women and men with prediabetes and type 2 diabetes had significantly higher levels of total fat and total lean mass and peripheral fat, trunk fat and gynoid fat mass (Table 2; Fig. 2b,d,f,j,l). Women with prediabetes and type 2 diabetes had significantly higher levels of peripheral lean mass than women with NGM. In men, this association was statistically significant only for prediabetes, not type 2 diabetes (Table 2; Fig. 2h). The differences in total and peripheral lean mass between type 2 diabetes and NGM, but not between prediabetes and NGM, were significantly greater in women than in men (women minus men [W–M] mean difference [95% CI]: 690 g [8, 1372] and 443 g [142, 744], respectively) (Table 2, model 3; Fig. 2d,h). The differences in total fat, peripheral fat, trunk fat and gynoid fat mass between type 2 diabetes and NGM and between prediabetes and NGM were not significantly different for women and men (Table 2, model 3; Fig. 2b,f,j,l).

**Table 2:** Differences within and between sexes in mean differences in measures of body composition according to glucose metabolism

Variable	Prediabetes β or GMR (95% CI)		Type 2 diabetes β or GMR (95% CI)		Sex difference WM-β or WM-GMR (95% CI)	
	Women	Men	Women	Men	Prediabetes	T2D
DEXA-derived measures of body composition						
Total body fat mass (g) (*n*=7639)						
Model 1	3665 (2855, 4476)	3497 (2703, 4291)	6502 (5691, 7313)	5689 (5025, 6354)	169 (−985, 1322)	813 (−240, 1865)
Model 2	3042 (2242, 3841)	2996 (2221, 3771)	5045 (4226, 5865)	4640 (3986, 5294)	46 (−1087, 1179)	405 (−655, 1465)
Model 3	2853 (2059, 3647)	2698 (1927, 3469)	4413 (3570, 5257)	4064 (3405, 4724)	155 (−969, 1278)	349 (−725, 1423)
Total body lean mass (g) (*n*=7639)						
Model 1	2147 (1643, 2651)	2144 (1639, 2649)	3773 (3270, 4275)	3250 (2828, 3673)	3 (−722, 728)	523 (−144, 1189)
Model 2	1010 (553, 1466)	726 (259, 1193)	1763 (1261, 2265)	964 (558, 1370)	283 (−382, 948)	799 (120, 1478)*
Model 3	1007 (550, 1464)	773 (305, 1241)	1751 (1250, 2251)	1060 (643, 1478)	234 (−431, 899)	690 (8, 1372)*
Peripheral fat mass (g) (*n*=7639)						
Model 1	1020 (649, 1391)	1118 (774, 1462)	1808 (1439, 2176)	1693 (1402, 1984)	−98 (−618, 422)	115 (−357, 586)
Model 2	753 (390, 1116)	927 (591, 1262)	1136 (774, 1499)	1270 (980, 1559)	−174 (−683, 336)	−133 (−597, 330)
Model 3	689 (326, 1052)	822 (487, 1157)	922 (544, 1300)	1068 (777, 1359)	−133 (−643, 376)	−146 (−624, 333)
Peripheral lean mass (g) (*n*=7639)						
Model 1	794 (549, 1039)	742 (497, 986)	1349 (1111, 1588)	853 (657, 1049)	52 (−298, 403)	496 (190, 803)*
Model 2	324 (103, 545)	221 (−7, 449)	515 (276, 754)	22 (−176, 219)	102 (−216, 421)	493 (193, 793)*
Model 3	330 (109, 552)	258 (29, 487)	541 (298, 784)	98 (−101, 298)	72 (−248, 392)	443 (142, 744)*
Trunk fat mass (g) (*n*=7639)						
Model 1	2641 (2144, 3138)	2312 (1842, 2781)	4685 (4199, 5171)	3923 (3545, 4300)	329 (−337, 996)	763 (152, 1373)*
Model 2	2275 (1787, 2763)	2006 (1546, 2465)	3880 (3389, 4371)	3299 (2920, 3679)	269 (−381, 919)	581 (−32, 1193)
Model 3	2149 (1665, 2633)	1822 (1366, 2278)	3459 (2973, 3945)	2944 (2562, 3325)	327 (−316, 971)	515 (−94, 1125)
Gynoid fat mass (g) (*n*=7639)						
Model 1	327 (202, 452)	395 (279, 510)	496 (378, 615)	554 (463, 646)	−67 (−238, 104)	−58 (−204, 88)
Model 2	241 (118, 364)	327 (213, 441)	296 (175, 416)	420 (328, 512)	−86 (−256, 84)	−124 (−272, 23)
Model 3	221 (99, 344)	291 (177, 405)	230 (108, 351)	349 (258, 441)	−69 (−239, 101)	−120 (−269, 29)
MRI-derived measures of body composition						
SAT (cm^2^) (*n*=7639)						
Model 1	30.6 (20.9, 40.4)	20.5 (11.9, 29.1)	52.3 (42.0, 62.5)	29.1 (21.4, 36.9)	10.2 (−2.5, 22.9)	23.1 (10.3, 35.9)*
Model 2	24.6 (15.0, 34.3)	17.6 (9.1, 26.0)	38.0 (27.6, 48.5)	21.3 (13.5, 29.0)	7.1 (−5.5, 19.6)	16.7 (3.7, 29.8)*
Model 3	22.7 (13.1, 32.4)	15.2 (6.7, 23.6)	31.6 (20.9, 42.3)	16.7 (8.6, 24.7)	7.5 (−5.1, 20.2)	15.0 (1.5, 28.5)*
VAT (cm^2^) (*n*=7639)						
Model 1	30.0 (21.4, 38.6)	37.8 (29.0, 46.5)	53.9 (45.1, 62.7)	72.1 (64.1, 80.0)	−7.8 (−19.9, 4.4)	−18.1 (−29.7, −6.6)*
Model 2	26.1 (17.6, 34.6)	31.4 (22.7, 40.0)	46.0 (37.3, 54.7)	61.7 (53.9, 69.5)	−5.3 (−17.3, 6.7)	−15.8 (−27.2, −4.3)*
Model 3	24.4 (16.0, 32.9)	28.0 (19.4, 36.6)	40.4 (31.6, 49.2)	55.2 (47.2, 63.2)	−3.6 (−15.5, 8.3)	−14.8 (−26.4, −3.1)*
Liver fat percentage (%)^a^ (*n*=7639)						
Model 1	1.39 (1.29, 1.50)	1.37 (1.26, 1.49)	1.71 (1.60, 1.83)	1.59 (1.49, 1.71)	1.04 (0.93, 1.17)	1.08 (0.97, 1.20)
Model 2	1.37 (1.26, 1.49)	1.28 (1.20, 1.38)	1.59 (1.46, 1.74)	1.55 (1.47, 1.64)	1.04 (0.94, 1.15)	1.03 (0.94, 1.13)
Model 3	1.35 (1.24, 1.47)	1.30 (1.20, 1.42)	1.52 (1.38, 1.66)	1.48 (1.38, 1.60)	1.03 (0.91, 1.16)	1.02 (0.91, 1.15)
Other anthropometric variable						
Hip circumference (cm) (*n*=7639)						
Model 1	3.7 (2.9, 4.4)	3.0 (2.3, 3.8)	9.0 (8.3, 9.7)	4.9 (4.3, 5.4)	0.6 (−0.5, 1.7)	4.1 (3.2, 5.1)*
Model 2	3.0 (2.2, 3.8)	2.7 (2.0, 3.4)	7.5 (6.7, 8.2)	4.1 (3.5, 4.7)	0.3 (−0.7, 1.4)	3.3 (2.4, 4.3)*
Model 3	2.9 (2.1, 3.6)	2.5 (1.8, 3.3)	7.0 (6.2, 7.7)	3.8 (3.2, 4.4)	0.3 (−0.7, 1.4)	3.2 (2.2, 4.1)*

Sex-specific differences are expressed as linear regression coefficients (95% CIs) of the dependent variables, indicating mean differences (βs) in fat mass, lean mass, SAT, VAT or hip circumference, or GMRs for liver fat percentage, according to GMS. The reference category for prediabetes and type 2 diabetes is normal GMS

Differences between sexes are expressed as linear regression coefficients (95% CIs) of the interaction terms sex-by-prediabetes and sex-by-type 2 diabetes, indicating differences between women and men in mean differences (WM-βs) in fat mass, lean mass, SAT, VAT or hip circumference or in GMRs (WM-GMRs) for liver fat percentage, according to GMS

Model 1: adjusted for age and height. Associations with liver fat percentage were not adjusted for height

Model 2: additionally adjusted for physical activity, healthy diet score, educational level, alcohol consumption and smoking status. Associations with total and peripheral lean mass were additionally adjusted for total fat mass

Model 3: additionally adjusted for the use of medication known to cause weight gain and/or loss as a possible side effect

For each potential confounder included, an interaction term (sex-by-potential confounder) was incorporated in the same model

^a^GMRs or WM-GMRs

*p<0.05

### MRI-derived measures of body composition

#### Sex as determinant

Women, independent of GMS, had significantly higher levels of SAT than men (Fig. 2m). In contrast men, independent of GMS, had significantly higher levels of VAT than women (Fig. 2o). Men with NGM, but not with prediabetes or type 2 diabetes, had a significantly higher liver fat percentage than women (Fig. 2q).

#### Sex-by-(pre)diabetes interaction

Both women and men with prediabetes and type 2 diabetes had significantly higher levels of SAT and VAT and a significantly higher liver fat percentage than their NGM counterparts (Table 2; Fig. 2n,p,r). The difference in SAT between type 2 diabetes and NGM, but not between prediabetes and NGM, was significantly greater in women than in men (W–M mean difference [95% CI]: 15.0 cm^2^ [1.5, 28.5]) (Table 2, model 3; Fig. 2n). The difference in VAT between type 2 diabetes and NGM, but not between prediabetes and NGM, was significantly greater in men than in women (W–M mean difference [95% CI]: −14.8 cm^2^ [−26.4, −3.1]) (Table 2, model 3; Fig. 2p). The differences in liver fat percentage between type 2 diabetes and NGM and between prediabetes and NGM were not significantly different between women and men (Table 2; Fig. 2r).

### Other anthropometric variable

#### Sex as determinant

Women, independent of GMS, had a significantly greater hip circumference than men (Fig. 2s).

#### Sex-by-(pre)diabetes interaction

Both women and men with prediabetes and type 2 diabetes, had a significantly greater hip circumference than their NGM counterparts, (Table 2; Fig. 2t). The difference in hip circumference between type 2 diabetes and NGM, but not between prediabetes and NGM, was significantly greater in women than in men (W–M mean difference [95% CI]: 3.2 cm [2.2, 4.1]) (Table 2; Fig. 2t).

In general, for all sex-by-prediabetes interactions investigated, the results of the more basic models (models 1 and 2) were comparable to those of the main model (model 3).

### Sensitivity analyses

After additional adjustment for DEXA and MRI lag time, the results did not materially change (ESM Table 1). In separate analyses of participants with a lag time ≤6 months, the greater differences in total lean mass and in SAT between type 2 diabetes and NGM in women than in men were attenuated (W–M mean difference [95% CI]: total lean mass from 690 g [8, 1372] to 559 g [−648, 1766], SAT from 15.0 cm^2^ [1.5, 28.5] to 5 cm^2^ [−16, 27]; ESM Table 2). The results of other analyses in participants with a lag time ≤6 months or >6 months were not materially different (ESM Table 2). Exclusion of premenopausal women (*n*=809) and women in whom menopausal status was unclear (*n*=69; analysis population *N*=6761) attenuated the greater difference in SAT between type 2 diabetes and NGM in women than in men (W–M mean difference [95% CI] from 15.0 cm^2^ [1.5, 28.5] to 11.2 cm^2^ [−3.0, 25.4]; ESM Table 3). Sex differences in hip circumference, VAT and lean mass did not materially change (ESM Table 3). Exclusion of participants with estimated values of SAT and VAT (*n*=250; analysis population *N*=4119) attenuated the greater difference in VAT between type 2 diabetes and NGM in men than in women (W–M mean difference [95% CI] from −11.2 cm^2^ [−24.2, 1.7] to −7.9 cm^2^ [−20.9, 5.1]; ESM Table 4). Sex differences in the results for SAT did not materially change (ESM Table 4). Complete case analysis (data not shown) gave similar sex differences to the multiple imputation approach. The statistical significance of the sex differences investigated differed for only two variables (i.e. trunk fat mass *p*=0.02 in the original dataset and *p*=0.10 in the imputed dataset; VAT *p*=0.09 in the original dataset and *p*=0.01 in the imputed dataset).

## Discussion

To our knowledge, this is the most comprehensive study to date that has investigated sex differences in body composition between people with prediabetes or type 2 diabetes and people with NGM. We showed that both women and men with prediabetes or type 2 diabetes had more fat mass, more lean mass and a greater hip circumference than their NGM counterparts. After adjustment for potential confounders, the differences in SAT and hip circumference between people with type 2 diabetes and people with NGM were greater in women than in men, whereas the difference in VAT was greater in men than in women. In addition, the difference in lean mass between those with type 2 diabetes and those with NGM was greater in women than in men. The differences in measures of body composition between those with prediabetes and those with NGM were generally in the same direction, but not statistically different for women and men.

In addition, women had more total, peripheral and gynoid fat mass than men, similar to previous findings [2, 16]. Women also had more trunk fat mass, for which previous findings have been inconsistent [17–19]. Regardless, we found that the larger amounts of total, peripheral, trunk and gynoid fat mass in people with type 2 diabetes than in those with NGM were not significantly different for women and men. These results suggest that, although women and men have different amounts of total, peripheral, trunk and gynoid fat mass, changes in the amounts that accompany the development of prediabetes and type 2 diabetes are similar among women and men.

Our results showed that women have more SAT and a greater hip circumference and men have more VAT, which is in line with previous studies [4, 20]. In addition, we observed that both women and men with prediabetes or type 2 diabetes had more SAT, a greater hip circumference and more VAT than their NGM counterparts, which is also in line with previous studies [21, 22].

The differences in SAT and hip circumference between people with type 2 diabetes and those with NGM were greater in women than in men. Excess body fat is associated with type 2 diabetes [23] and the observed sex differences in SAT and hip circumference could be explained by the preferential storage of excess body fat in subcutaneous and peripheral adipose tissue in women [4]. Excess body fat in these fat depots is considered less harmful than excess VAT [4, 24]. Moreover, subcutaneous thigh fat and gluteofemoral body fat have been associated with more favourable levels of glucose and lipids [25, 26] and a lower likelihood of the metabolic syndrome [26, 27]. Large hip and thigh circumferences have also been associated with a lower risk of type 2 diabetes [28]. However, the results regarding the sex difference in hip circumference should be interpreted carefully, as the difference in gynoid fat mass between people with type 2 diabetes and those with NGM was not significantly greater in women than in men.

In individuals with obesity, excessive amounts of VAT and related lipid accumulation in the liver and pancreas define the increased risk of type 2 diabetes and CVD [23, 29]. However, our results imply that this process differs between women and men. We observed that the difference in VAT between people with type 2 diabetes and those with NGM was greater in men than in women, but the difference in liver fat percentage was not statistically different between women and men. This may imply that women developing type 2 diabetes have a similar increase in liver fat despite a smaller increase in VAT than men. These differences might be explained by women’s greater increase in SAT during the transition to type 2 diabetes, as implied by our results. SAT can be divided into two layers: superficial and deep SAT [30]. Deep SAT is thought to be more metabolically harmful than superficial SAT [31], but whether any harmful effects of superficial and deep SAT differ between men and women is not clear [30, 32]. Taken together, with increasing weight, women may have a smaller increase in VAT but a higher increase in SAT than men. Because of the harmful effects of deep SAT, this might have a similar adverse effect on liver fat deposition and the development of type 2 diabetes. Alternatively, or additionally, VAT may also have sex-specific effects on diabetes development. VAT seems to have a stronger association with metabolic risk factors [33] and insulin resistance [34] in women than in men. Thus, although women may have a smaller increase in VAT, this does not necessarily indicate that it is less detrimental for diabetes development. In our data we did not distinguish between superficial and deep SAT. The sex-specific role of SAT and VAT in diabetes development requires further investigation. If the observed sex differences are important for the pathogenesis of type 2 diabetes, sex-specific prevention measures may be necessary.

In general, our results indicate that, in diabetes, women have more total fat mass than men, which is distributed differently, that is, more peripheral, trunk and gynoid fat mass, a larger hip circumference, more SAT and less VAT and a similar liver fat percentage. Furthermore, our results imply that, during the transition to type 2 diabetes, women have greater increases in SAT and hip circumference and a smaller increase in VAT, but a similar increase in liver fat.

We attribute the attenuated greater difference in lean mass and SAT between type 2 diabetes and NGM in women than in men in participants having a DEXA and MRI lag time ≤6 months to chance and loss of power. We measured weight at baseline and also at the time of the DEXA scan. We have no information on weight at the time of the MRI scan. For men, the difference in weight at the time of the DEXA scan compared with baseline was 0.5 kg (mean ± SD 87.0 ± 14.3 vs 86.5 ± 13.8). For women, the difference was 0.4 kg (mean ± SD 72.0 ± 13.7 vs 71.6 ± 13.4). This weight difference is probably comparable to that which would have been observed at the time of the MRI scan compared with baseline. The small differences in weight are unlikely to have affected the results and indeed most results in this sensitivity analysis were not materially different.

The exclusion of premenopausal women in the additional analyses attenuated the greater difference in SAT between type 2 diabetes and NGM in women than in men. This was a result of the smaller mean difference in SAT between postmenopausal women with type 2 diabetes and postmenopausal women with NGM, which can be explained by a decrease in oestrogen levels after the menopause. Oestrogen favours the deposition of SAT and decreased oestrogen levels lead to a smaller proportion of fat gain in SAT [4].

For men and women who had a body size that prohibited the direct determination of the complete amounts of VAT and SAT, estimated values were used. The exclusion of participants with estimated values of SAT and VAT attenuated the greater difference in VAT between type 2 diabetes and NGM in men than in women. As these participants generally had larger amounts of VAT and were not missing at random, range restriction could explain the observed attenuated sex difference [35].

We observed that men had more lean mass than women, which is in line with previous research [2]. We additionally observed that both women and men with prediabetes or type 2 diabetes had more lean mass than their NGM counterparts and that the difference in lean mass between type 2 diabetes and NGM was greater in women than in men. Previous data on diabetes-associated lean mass have been inconsistent [7–10, 36]. Hyperglycaemia and type 2 diabetes have been associated with lower levels of lean mass [7, 8, 36]; however, it has also been suggested that high levels of lean mass do not protect against insulin resistance [9, 10]. Although we adjusted for fat mass, residual effects of adiposity may underlie the positive associations of prediabetes and type 2 diabetes with lean mass. Increased adiposity has been suggested to act as a chronic overload stimulus on the muscles, increasing muscle size and strength [37]. A further possibility is that, in people with increased adiposity and type 2 diabetes, lean mass is less functional because of skeletal muscle lipid infiltration [10]. In turn, lean mass has to increase to compensate for malfunction. Whether women are more susceptible to these mechanisms is unclear. Nevertheless, women’s greater difference in lean mass between type 2 diabetes and NGM does not seem to be explained by differences in lifestyle factors, as we previously found that there were no sex differences in the association of type 2 diabetes with physical activity and healthy diet score [38].

The strengths of our study include its population-based design combined with oversampling of individuals with type 2 diabetes, which enabled an accurate comparison of individuals with and without type 2 diabetes. Additionally, this study benefits from the large sample size and the detailed phenotypic assessment. There are also some limitations. First, the data were cross-sectional; therefore, we cannot determine the causality and direction in the associations of measures of body composition and (pre)diabetes. However, we do not expect this to affect the sex differences investigated. Second, our population was generally relatively healthy; this may have resulted in an underestimation of the sex-specific associations found and the differences therein. Additionally, our study population consisted of middle-aged white individuals. Our results are generalisable to individuals with similar characteristics, but it should be kept in mind that the associations and sex differences found may differ in populations with a different distribution of determinants or in other ethnic groups. Third, some variables had missing data. After multiple imputation, the observed sex differences were comparable to those found in the complete case analysis. Fourth, the interplay of sex, body composition and type 2 diabetes is complex. We did not investigate sex differences in the associations of measures of body composition with insulin resistance or beta cell function, or sex differences in the association of pancreatic fat with (pre)diabetes, which may aid our understanding of sex differences in the role of body composition in the development of type 2 diabetes, as this was beyond the scope of this study. Finally, the percentage of women in the type 2 diabetes study population was about 14 percentage points lower than that in the source population [39]. If the apparent under-representation of women with type 2 diabetes was the result of health selection, this could have influenced the sex differences seen. However, the recruitment strategy was the same for women and men.

### Conclusion

In conclusion, we found that differences in SAT, hip circumference and lean mass between people with type 2 diabetes and people with NGM were greater in women than in men, and the difference in VAT was greater in men than in women. There was no difference in the percentage of liver fat between people with type 2 diabetes and people with NGM. These results suggest that there is a sex-specific role of body composition in the development of type 2 diabetes and that sex-specific prevention measures are necessary.

## Supplementary Information

ESM (PDF 695 kb)

## Data Availability

The datasets generated during and/or analysed during the current study are available from the corresponding author on reasonable request.

## Abbreviations

DEXA: Dual-energy x-ray absorptiometry
GMR: Geometric mean ratio
GMS: Glucose metabolism status
NGM: Normal glucose metabolism
SAT: Subcutaneous adipose tissue
VAT: Visceral adipose tissue
W–M: Women minus men
