# Supplementary material for: Sex differences in body composition in people with prediabetes and type 2 diabetes as compared with people with normal glucose metabolism: the Maastricht Study
Source: Diabetologia. 2023 Feb 20;66(5):861–72. doi: 10.1007/s00125-023-05880-0 (PMC10036428; doi:10.1007/s00125-023-05880-0)
Supplement: Supplementary file 1 — (PDF 695 kb) [file 125_2023_5880_MOESM1_ESM.pdf]

Electronic supplementary material (ESM)

ESM – methods

Assessment of body composition

A Hologic DEXA scanner, type discovery, was used to assess the amount of fat and lean mass (g), as described in more detail elsewhere (1). A subdivision was made to differentiate between storage locations of fat mass (total body, trunk, gynoid and peripheral region (i.e. upper and lower extremities; Supplemental Figure 1)) and lean mass (i.e. fat-free soft tissue mass; total body and peripheral region (i.e. upper and lower extremities; Supplemental Figure 1)).

MRI imaging was performed using a 3.0 Tesla MRI system (MAGNETOM Prismafit, Siemens Healthineers, Erlangen, Germany) using the body matrix and supine radiofrequency coils. Subjects were positioned supine. After scout scans, a T1-weighted image was obtained to quantify abdominal adipose tissue area. Subcutaneous adipose tissue (SAT) and visceral adipose tissue (VAT) (cm^2) were determined at the top level of the fourth lumbar vertebral body using a single-slice turbo spin echo MR image in the transverse plane that was acquired during a breath-hold (Supplemental Figure 1). The acquisition parameters were: echo time = 35-38 ms, repetition time = 550 ms, field-of-view = 384-500 x 312-406 mm^2, slice thickness = 8 mm, acquired voxel size = 2.0×2.0×8.0 mm, and GRAPPA with a reduction factor of 2. It has been previously demonstrated that single-slice MR images at the level of the fourth lumbar vertebra are accurate for representing the total amount of SAT and VAT (Pearson's $r \geq 0.89$) (2). SAT and VAT was quantified by trained observers using dedicated semi-automatic software (Quantib Abdominal Segmentation Tool [QAST], Quantib, Rotterdam, the Netherlands) based on a Gaussian curve fit to distinguish between fatty and non-fatty tissue with manual review of each segmentation analysis. The algorithm used by QAST is identical to the validated Hippofat software (3-5), yet allows for more efficient data handling and analysis of liver fat within one software environment.

Because of a technical error (insufficient field of view on the first MR images) in 250 participants, the amount of SAT (n=250) and VAT (n=28) was inadvertently incomplete. In order to estimate the SAT and VAT in these participants as accurately as possible, a subset of participants with the highest waist circumference, yet without this issue was used to estimate (missing) SAT and VAT volume. In this subset of participants with complete MRI data, the amount of missing SAT and VAT was modeled based on age, sex and waist circumference, as well as the amount of VAT and SAT that was visible with incomplete settings. Herewith, actual SAT (ICC 0.978 (95%-CI 0.974; 0.982), n=433) and VAT (ICC 0.949 (0.901-0.974) n=35) could be estimated accurately.

Liver fat percentage was assessed through Dixon MR imaging (Supplemental Figure 1), as described in more detail elsewhere (6). In brief, after a scout scan, transversal two-dimensional T2-weighted True Fast Imaging with steady-state-free precession (T2w TRUFI) images were acquired through the liver. Next, transversal two-dimensional turbo spin echo Dixon MR images were acquired through the liver during a breathhold. Three regions-of-interest (ROIs) were drawn on the T2w TRUFI images of the liver by trained observers. Care was taken to position these ROIs avoiding visible structures in the liver, such as vessels and bile ducts, and in artifact-free regions. Subsequently, these ROIs were copied to the water and fat Dixon MR images to calculate the intrahepatic lipid fraction. This method was validated and calibrated against proton magnetic resonance spectroscopy (^{1}H-MRS), the gold standard to non-invasively quantify IHL, in 36 participants. With ^{1}H-MRS the CH_2/H_2O ratio was determined by calculating the ratio of the T_2-corrected spectral peak areas of CH_2 and water. Liver fat percentage was expressed as the ratio CH_2/H_2O (*100%). After calibration, the intra-class correlation coefficient between Dixon MRI and ^{1}H-MRS was 0.989 (95% CI: 0.979; 0.994).

Assessment of glucose metabolism status

To determine glucose metabolism status (GMS), all participants underwent a standardized 2-hour (2-h) 75 gram oral glucose tolerance test after fasting overnight. For safety reasons, participants using insulin or with a fasting glucose level above 11.0 mmol/L, as determined by a finger prick, did not undergo the oral glucose tolerance test. For these individuals, fasting glucose level and information about diabetes medication were used to determine GMS. GMS was defined according to the WHO 2006 criteria into normal glucose metabolism, impaired fasting glucose, impaired glucose tolerance (combined as prediabetes), and type 2 diabetes (7). Participants on blood glucose lowering medication were classified as having type 2 diabetes.

Assessment of covariates and population characteristics

We used a questionnaire to assess age (years), sex, smoking status (never, current, former), alcohol use (g/day), adherence to the Dutch dietary guidelines and the indication of diet quality (based on fourteen out of fifteen components of the Dutch Healthy Diet index 2015, as information on filtered coffee intake was not collected (8)), calculated from a validated food frequency questionnaire (9) the total score ranges between 0 (no adherence) and 130 (complete adherence), educational level (low, intermediate, high), physical activity level (hours of moderate to vigorous physical activity per week) and postmenopausal status in women (10). Women who indicated that they had had a menstrual period in the preceding 12 months were classified as premenopausal women; if they indicated that they had not had a menstrual period in the preceding 12 months they were classified as postmenopausal women. We assessed medication use, e.g. glucose-lowering, lipid-modifying and antihypertensive medication use, as well as postmenopausal hormone replacement therapy, during a medication interview where generic name, dose, and frequency were registered (10). Additionally, a variable was

constructed which included medication known for weight gain or weight loss as possible side effect. It was defined as using one or more of the following drugs: hormonal contraceptives, antidepressants, antipsychotic drugs, lithium, medicinal cannabis, beta-blockers, anti-epileptics (i.e. valproic acid, gabapentin, carbamazepine and topiramate) mineralocorticoids (i.e. fludrocortisone), glucocorticoids (i.e. betamethasone, dexamethasone, methylprednisolone, prednisolone, prednisone, triamcinolone (acetonide), hydrocortisone or cortisone) (11). We determined HbA1c, fasting glucose, 2-h postload glucose, waist and hip circumference, BMI, triglyceride levels, total cholesterol, HDL cholesterol and systolic and diastolic office blood pressure as described elsewhere(10).

Statistical analyses

To investigate the robustness of the results obtained by the described analyses, we did several sensitivity analyses. In the imputed dataset (N=7639), a sensitivity analysis was conducted to investigate if time between baseline measurements and the DEXA and MRI scan (lag time), which were performed at a later moment in time, influenced the results. We first adjusted for lag time and additionally we repeated the original analyses in participants having a lag time ≤ 6 months (DEXA N=2129, MRI N=2834) and in participants having a lag time > 6 months (DEXA N=5510, MRI N=4805). Second, we repeated all analyses after exclusion of premenopausal women (N=809) and women in whom menopausal status was unclear (N=69; analysis population N=6761). Third, if body size prohibited the determination of the amount of SAT and VAT according to the study protocol, estimated values were used. We repeated the analyses after exclusion of participants with estimated values (N=250; analysis population N=4119) in the original (not imputed) dataset. We performed these analyses in the original dataset as the variables of SAT and VAT, including the estimated values, were used for imputation of the data. For these analyses, complete case analyses were performed. Finally, for

all investigated associations, we performed complete case analysis in the original dataset to compare the results with the multiple imputation approach.

References

1. Applicatiehandleiding Hologic Discovery; Hologic nv Leuvensesteenweg 250 A 1800 Vilvoorde; Appssupport.ROW@hologic.com; https://www.hologic.nl/.
2. Schweitzer L, Geisler C, Pourhassan M, Braun W, Gluer CC, Bosy-Westphal A, et al. What is the best reference site for a single MRI slice to assess whole-body skeletal muscle and adipose tissue volumes in healthy adults? Am J Clin Nutr. 2015;102(1):58-65.
3. Postinato, J Magn Reson Imaging. 2004; 20(4):684-689.
4. Postinato, J Magn Reson Imaging. 2008; 28:403-410.
5. Postinato, Proc. Intl. Soc. Mag. Reson. Med. 2009; 17; 4086.
6. Brouwers M, Simons N, Kooi ME, de Ritter R, van Dongen M, Eussen S, et al. Intrahepatic lipid content is independently associated with soluble E-selectin levels: The Maastricht study. Dig Liver Dis. 2022.
7. World Health Organization & International Diabetes Federation. (2006). Definition and diagnosis of diabetes mellitus and intermediate hyperglycaemia : report of a WHO/IDF consultation. World Health Organization. https://apps.who.int/iris/handle/10665/43588.
8. Looman M, Feskens EJ, de Rijk M, Meijboom S, Biesbroek S, Temme EH, et al. Development and evaluation of the Dutch Healthy Diet index 2015. Public Health Nutr. 2017;20(13):2289-99.
9. van Dongen MC, Wijckmans-Duysens NEG, den Biggelaar LJ, Ocke MC, Meijboom S, Brants HA, et al. The Maastricht FFQ: Development and validation of a comprehensive food frequency questionnaire for the Maastricht study. Nutrition. 2018;62:39-46.
10. Schram MT, Sep SJ, van der Kallen CJ, Dagnelie PC, Koster A, Schaper N, et al. The Maastricht Study: an extensive phenotyping study on determinants of type 2 diabetes, its complications and its comorbidities. Eur J Epidemiol. 2014;29(6):439-51.
11. Apotheek.nl Alles over medicijnen. Betrouwbare informatie van de apotheker. https://www.apotheek.nl/zorg-van-de-apotheker/gestart-met-uw-medicijnen-wat-nu/gewichtstoename-bij-medicijngebruik#medicijnen-die-gewichtstoename-kunnen-veroorzaken.

ESM Table 1 Differences between sexes in mean differences in measures of body composition according to glucose metabolism – additional adjustment (model 4) for DEXA lag time (for DEXA-derived variables) and MRI lag time (for MRI-derived variables).

	Sex difference<br>WM-β or WM-GMR (95%-CI)<br>Prediabetes	T2DM
DEXA-derived measures of body composition		
Total body fat mass (g)<br>n=7639 *Model 3*<br>*Model 4*	155 (-969;1278)<br>158 (-966;1282)	349 (-725;1423)<br>359 (-716;1435)
Total body lean mass (g)<br>n=7639 *Model 3*<br>*Model 4*	234 (-431;899)<br>231 (-433;895)	690 (8;1372)*<br>677 (-6;1361)*
Peripheral fat mass (g)<br>n=7639 *Model 3*<br>*Model 4*	-133 (-643;376)<br>-132 (-642;378)	-146 (-624;333)<br>-142 (-622;337)
Peripheral lean mass (g)<br>n=7639 *Model 3*<br>*Model 4*	72 (-248;392)<br>70 (-251;391)	443 (142;744)*<br>436 (133;739)*
Trunk fat mass (g)<br>n=7639 *Model 3*<br>*Model 4*	327 (-316;971)<br>329 (-315;973)	515 (-94;1125)<br>523 (-88;1134)
Gynoid fat mass (g)<br>n=7639 *Model 3*<br>*Model 4*	-69 (-239;101)<br>-69 (-239;101)	-120 (-269;29)<br>-119 (-268;30)
MRI-derived measures of body composition		
Subcutaneous adipose tissue (cm^2) n=7639<br>*Model 3*<br>*Model 4*	7.5 (-5.1;20.2)<br>7.4 (-5.2;20.0)	15.0 (1.5;28.5)*<br>14.8 (1.3;28.4)*
Visceral adipose tissue (cm^2) n=7639<br>*Model 3*<br>*Model 4*	-3.6 (-15.5;8.3)<br>-3.6 (-15.5;8.3)	-14.8 (-26.4;-3.1)*<br>-14.7 (-26.3;-3.0)*
Fat percentage liver (%)[a]<br>n=7639 *Model 3*<br>*Model 4*	1.03 (0.92;1.16)<br>1.03 (0.92;1.16)	1.02 (0.91;1.15)<br>1.02 (0.91;1.14)

DEXA=dual-energy X-ray absorptiometry; MRI=magnetic resonance imaging.

Sex-specific differences are expressed as linear regression coefficients (95%-CI) of the dependent variables, which indicate mean differences (βs) or geometric mean ratios (GMRs) in amount of fat mass, lean mass, subcutaneous or visceral adipose tissue or liver fat percentage according to glucose metabolism status. The reference category for prediabetes and type 2 diabetes is normal glucose metabolism status. [a] GMRs

Differences between sexes are expressed as linear regression coefficients (95%-CI) of the interaction terms sex*prediabetes and sex*type 2 diabetes, which indicate differences between women and men in mean differences (WM-βs) or women to men ratio of geometric mean ratios (WM-GMRs) in amount of fat mass, lean mass, subcutaneous or visceral adipose tissue or liver fat percentage according to glucose metabolism status. [a]WM-GMRs. Statistically significant differences between the sexes are typed in bold. *P value <0.05

Model 3: adjusted for age, height, physical activity, healthy diet score, educational level, alcohol consumption and smoking status and use of medication known for weight gain and/or loss as possible side effect†

Model 4: additionally adjusted for DEXA lag time (for DEXA-derived variables) and MRI lag time (for MRI-derived variables)

For each potential confounder included, an interaction term (sex by potential confounder) was incorporated in the same model

† associations with liver fat percentage were not adjusted for height; associations with total and peripheral lean mass were additionally adjusted for total fat mass

ESM Table 2 Differences between sexes in mean differences in measures of body composition according to glucose metabolism – results (model 3) presented separately for participants with a lag time ≤0.5 years and >0.5 years (DEXA lag time for DEXA-derived variables and MRI lag time for MRI-derived variables).

	Sex difference<br>WM-β or WM-GMR (95%-CI)<br>Prediabetes	 <br>T2DM
DEXA-derived measures of body composition		
Total body fat mass (g)<br>*Model 3 (total) n=7639*<br>*Model 3 (lagtime ≤ 0.5y) n=2129*<br>*Model 3 (lagtime >0.5y) n=5510*	155 (-969;1278)<br>-1359 (-3388;669)<br>759 (-571;2088)	349 (-725;1423)<br>154 (-1735;2044)<br>403 (-873;1679)
Total body lean mass (g) n=7639<br>*Model 3 (total) n=7639*<br>*Model 3 (lagtime ≤ 0.5y) n=2129*<br>*Model 3 (lagtime >0.5y) n=5510*	234 (-431;899)<br>-753 (-1992;486)<br>610 (-192;1411)	690 (8;1372)*<br>559 (-648;1766)<br>710 (-103;1524)
Peripheral fat mass (g) n=7639<br>*Model 3 (total) n=7639*<br>*Model 3 (lagtime ≤ 0.5y) n=2129*<br>*Model 3 (lagtime >0.5y) n=5510*	-133 (-643;376)<br>-551 (-1511;409)<br>28 (-564;621)	-146 (-624;333)<br>-390 (-1244;463)<br>-71 (-658;515)
Peripheral lean mass (g) n=7639<br>*Model 3 (total) n=7639*<br>*Model 3 (lagtime ≤ 0.5y) n=2129*<br>*Model 3 (lagtime >0.5y) n=5510*	72 (-248;392)<br>-311 (-921;299)<br>220 (-152;592)	443 (142;744)*<br>434 (-161;1029)<br>437 (70;804)*
Trunk fat mass (g)<br>*Model 3 (total) n=7639*<br>*Model 3 (lagtime ≤ 0.5y) n=2129*<br>*Model 3 (lagtime >0.5y) n=5510*	327 (-316;971)<br>-649 (-1890;593)<br>720 (-35;1475)	515 (-94;1125)<br>508 (-754;1770)<br>507 (-222;1237)
Gynoid fat mass (g)<br>*Model 3 (total) n=7639*<br>*Model 3 (lagtime ≤ 0.5y) n=2129*<br>*Model 3 (lagtime >0.5y) n=5510*	-69 (-239;101)<br>-298 (-605;9)<br>21 (-176;219)	-120 (-269;29)<br>-220 (-521;82)<br>-84 (-265;98)
MRI-derived measures of body composition		
Subcutaneous adipose tissue (cm^2) n=7639<br>*Model 3 (total) n=7639*<br>*Model 3 (lagtime ≤ 0.5y) n=2834*<br>*Model 3 (lagtime >0.5y) n=4805*	7.5 (-5.1;20.2)<br>-4 (-29;20)<br>14 (-3;32)	15.0 (1.5;28.5)*<br>5 (-16;27)<br>20 (4;37)*
Visceral adipose tissue (cm^2)<br>*Model 3 (total) n=7639*<br>*Model 3 (lagtime ≤ 0.5y) n=2834*<br>*Model 3 (lagtime >0.5y) n=4805*	-3.6 (-15.5;8.3)<br>-9.5 (-29.6;10.7)	-14.8 (-26.4;-3.1)*<br>-14.2 (-31.9;3.6)

	0.7 (-14.4;15.8)	-14.6 (-30.1;1.0)
Fat percentage liver (%)[a]<br>*Model 3 (total) n=7639*<br>*Model 3 (lagtime ≤ 0.5y) n=2834*<br>*Model 3 (lagtime >0.5y) n=4805*	 1.03 (0.92;1.16)<br>0.93 (0.76;1.13)<br>1.10 (0.95;1.28)	 1.02 (0.91;1.15)<br>0.99 (0.81;1.22)<br>1.05 (0.91;1.21)

DEXA=dual-energy X-ray absorptiometry; MRI=magnetic resonance imaging.

Differences between sexes are expressed as linear regression coefficients (95%-CI) of the interaction terms sex*prediabetes and sex*type 2 diabetes, which indicate differences between women and men in mean differences (WM-βs) or women to men ratio of geometric mean ratios (WM-GMRs) in amount of fat mass, lean mass, subcutaneous or visceral adipose tissue or liver fat percentage according to glucose metabolism status. †WM-GMRs. Statistically significant differences between the sexes are typed in bold. *P value <0.05

Model 3: adjusted for age, height, physical activity, healthy diet score, educational level, alcohol consumption and smoking status and use of medication known for weight gain and/or loss as possible side effect†

For each potential confounder included, an interaction term (sex by potential confounder) was incorporated in the same model

† associations with liver fat percentage were not adjusted for height; associations with total and peripheral lean mass were additionally adjusted for total fat mass

ESM Table 3 Differences within and between sexes in mean differences in measures of body composition according to glucose metabolism – exclusion of premenopausal women

	Prediabetes β or GMR (95%-CI)		Type 2 diabetes β or GMR (95%-CI)		Sex difference WM-β or WM-GMR (95%-CI)	
	Women	Men	Women	Men	Prediabetes	T2DM
DEXA-derived measures of body composition						
Total body fat mass (g)<br>n=6761 *Model 1*<br>*Model 2*<br>*Model 3*	3421 (2521;4321)<br>2789 (1899;3679)<br>2584 (1700;3468)	3497 (2708;4285)<br>2996 (2227;3765)<br>2698 (1934;3462)	6259 (5387;7130)<br>4719 (3845;5593)<br>4108 (3203;5013)	5689 (5029;6349)<br>4640 (3991;5289)<br>4064 (3410;4719)	-75 (-1298;1147)<br>-207 (-1410;997)<br>-114 (-1306;1077)	569 (-530;1668)<br>79 (-1023;1181)<br>28 (-1089;1144)
Total body lean mass (g)<br>n=6761 *Model 1*<br>*Model 2*<br>*Model 3*	2077 (1512;2641)<br>1016 (501;1530)<br>1019 (505;1533)	2144 (1635;2652)<br>726 (256;1196)<br>773 (301;1244)	3584 (3015;4152)<br>1651 (1102;2201)<br>1664 (1120;2208)	3250 (2825;3676)<br>964 (555;1373)<br>1060 (640;1481)	-67 (-844;710)<br>289 (-425;1004)<br>247 (-468;961)	333 (-389;1055)<br>687 (-34;1409)<br>604 (-119;1327)
Peripheral fat mass (g)<br>n=6761 *Model 1*<br>*Model 2*<br>*Model 3*	953 (548;1359)<br>690 (293;1086)<br>617 (220;1014)	1118 (780;1456)<br>927 (597;1256)<br>822 (494;1150)	1765 (1360;2169)<br>1051 (660;1443)<br>830 (426;1234)	1693 (1407;1979)<br>1270 (985;1555)<br>1068 (782;1353)	-165 (-705;375)<br>-237 (-767;294)<br>-205 (-735;326)	72 (-423;566)<br>-218 (-700;263)<br>-238 (-731;255)
Peripheral lean mass (g)<br>n=6761 *Model 1*<br>*Model 2*<br>*Model 3*	733 (456;1010)<br>304 (50;557)<br>314 (60;567)	742 (496;988)<br>221 (-9;452)<br>258 (27;489)	1242 (981;1504)<br>453 (193;713)<br>488 (223;753)	853 (656;1051)<br>22 (-177;221)<br>98 (-103;299)	-9 (-385;368)<br>82 (-261;426)<br>55 (-289;400)	389 (65;713)*<br>432 (117;747)*<br>390 (74;706)*
Trunk fat mass (g)<br>n=6761 *Model 1*<br>*Model 2*<br>*Model 3*	2457 (1899;3015)<br>2077 (1531;2623)<br>1942 (1401;2483)	2312 (1842;2781)<br>2006 (1547;2464)<br>1822 (1367;2277)	4508 (3989;5028)<br>3659 (3138;4181)<br>3248 (2731;3764)	3923 (3545;4300)<br>3299 (2921;3678)<br>2944 (2563;3325)	145 (-563;853)<br>71 (-620;762)<br>120 (-563;803)	586 (-64;1236)<br>360 (-290;1010)<br>304 (-342;950)

Gynoid fat mass (g) n=6761 *Model 1* *Model 2* *Model 3*	395 (281;508)<br>327 (216;439)<br>291 (179;403)	290 (157;424)<br>205 (73;336)<br>182 (52;313)	554 (464;644)<br>420 (330;510)<br>349 (260;439)	480 (347;612)<br>270 (136;405)<br>202 (67;337)	-104 (-280;71)<br>-122 (-297;52)<br>-109 (-283;65)	-75 (-229;80)<br>-150 (-307;7)<br>-148 (-306;10)
MRI-derived measures of body composition						
Subcutaneous adipose tissue (cm^2) n=6761 *Model 1* *Model 2* *Model 3*	27.2 (16.1;38.3)<br>21.3 (10.2;32.3)<br>19.3 (8.3;30.3)	20.5 (12.1;28.9)<br>17.6 (9.3;25.9)<br>15.2 (6.8;23.5)	48.5 (37.5;59.4)<br>33.9 (22.6;45.3)<br>27.9 (16.4;39.3)	29.1 (21.5;36.7)<br>21.3 (13.6;28.9)<br>16.7 (8.8;24.6)	6.7 (-7.1;20.6)<br>3.7 (-10.0;17.4)<br>4.1 (-9.7;17.9)	19.4 (5.9;32.9)*<br>12.7 (-1.2;26.5)<br>11.2 (-3.0;25.4)
Visceral adipose tissue (cm^2) n=6761 *Model 1* *Model 2* *Model 3*	29.5 (19.7;39.4)<br>25.4 (15.7;35.0)<br>23.7 (14.1;33.3)	37.8 (28.8;46.7)<br>31.4 (22.5;40.2)<br>28.0 (19.2;36.8)	51.2 (41.3;61.1)<br>43.0 (33.3;52.7)<br>38.0 (28.2;47.7)	72.1 (64.0;80.1)<br>61.7 (53.8;69.7)<br>55.2 (47.0;63.4)	-8.2 (-21.5;5.1)<br>-6.0 (-19.2;7.1)<br>-4.3 (-17.3;8.8)	-20.8 (-33.3;-8.3)*<br>-18.7 (-31.0;-6.5)*<br>-17.2 (-29.7;-4.7)*
Fat percentage liver (%)[a] n=6761 *Model 1* *Model 2* *Model 3*	1.44 (1.31;1.57)<br>1.37 (1.25;1.50)<br>1.35 (1.23;1.48)	1.37 (1.26;1.49)<br>1.32 (1.21;1.43)<br>1.30 (1.20;1.42)	1.65 (1.49;1.84)<br>1.54 (1.39;1.70)<br>1.47 (1.33;1.64)	1.59 (1.49;1.71)<br>1.52 (1.41;1.64)<br>1.48 (1.38;1.60)	1.05 (0.93;1.18)<br>1.04 (0.92;1.17)<br>1.04 (0.92;1.17)	1.04 (0.92;1.17)<br>1.01 (0.90;1.14)<br>0.99 (0.88;1.13)
Other anthropometric variable						
Hip circumference (cm) n=6761 *Model 1* *Model 2* *Model 3*	3.6 (2.7;4.4)<br>2.9 (2.1;3.7)<br>2.8 (1.9;3.6)	3.0 (2.3;3.8)<br>2.7 (2.0;3.4)<br>2.5 (1.8;3.2)	9.0 (8.2;9.7)<br>7.3 (6.5;8.1)<br>6.8 (6.0;7.6)	4.9 (4.3;5.4)<br>4.1 (3.5;4.7)<br>3.8 (3.2;4.4)	0.6 (-0.6;1.7)<br>0.2 (-0.9;1.3)<br>0.2 (-0.9;1.3)	4.1 (3.1;5.1)*<br>3.2 (2.2;4.2)*<br>3.0 (2.0;4.0)*

DEXA=dual-energy X-ray absorptiometry; MRI=magnetic resonance imaging.

Sex-specific differences are expressed as linear regression coefficients (95%-CI) of the dependent variables, which indicate mean differences (βs) or geometric mean ratios (GMRs) in fat mass, lean mass, subcutaneous or visceral adipose tissue, liver fat percentage or hip circumference according to glucose metabolism status. The reference category for prediabetes and type 2 diabetes is normal glucose metabolism status. [a] GMRs

Differences between sexes are expressed as linear regression coefficients (95%-CI) of the interaction terms sex*prediabetes and sex*type 2 diabetes, which indicate differences between women and men in mean differences (WM-βs) or women to men ratio of geometric mean ratios (WM-GMRs) in fat mass, lean mass, subcutaneous or visceral adipose tissue, liver fat percentage or hip circumference according to glucose metabolism status. †WM-GMRs. Statistically significant differences between the sexes are typed in bold. *P value <0.05

Model 1: adjusted for age and height†

Model 2: additionally adjusted for physical activity, healthy diet score, educational level, alcohol consumption and smoking status‡

Model 3: additionally adjusted for the use of medication known for weight gain and/or loss as possible side effect

For each potential confounder included, an interaction term (sex by potential confounder) was incorporated in the same model

† associations with liver fat percentage were not adjusted for height

‡ associations with total and peripheral lean mass were additionally adjusted for total fat mass

ESM Table 4 Differences between sexes in mean differences in measures of body composition according to glucose metabolism – exclusion of participants with estimated values of SAT and VAT

	Sex difference WM-β (95%-CI) Prediabetes	T2DM
Group with exclusion of participants with estimated values of SAT and VAT		
Subcutaneous adipose tissue (cm^2) n=4119		
Model 1	6.2 (-8.2;20.5)	32.1 (18.4;45.7)*
Model 2	5.1 (-9.1;19.3)	29.3 (15.6;43.1)*
Model 3	5.0 (-9.2;19.1)	26.9 (13.0;40.7)*
Visceral adipose tissue (cm^2) n=4119		
Model 1	-10.7 (-24.3;2.9)	-10.6 (-23.5;2.3)
Model 2	-8.1 (-21.4;5.2)	-8.1 (-21.0;4.8)
Model 3	-7.2 (-20.4;6.0)	-7.9 (-20.9;5.1)
Total group		
Subcutaneous adipose tissue (cm^2) n=4369		
Model 1	10.9 (-3.7;25.4)	32.4 (18.7;46.1)*
Model 2	9.4 (-5.0;23.8)	28.9 (15.1;42.6)*
Model 3	9.6 (-4.8;23.9)	26.8 (12.9;40.8)*
Visceral adipose tissue (cm^2) n=4369		
Model 1	-11.6 (-25.2;2.1)	-15.4 (-28.2;-2.5)*
Model 2	-9.1 (-22.5;4.3)	-12.3 (-25.1;0.5)
Model 3	-7.8 (-21.1;5.6)	-11.2 (-24.2;1.7)

SAT=subcutaneous adipose tissue; VAT=visceral adipose tissue

Differences between sexes are expressed as linear regression coefficients (95%-CI) of the interaction terms sex*prediabetes and sex*type 2 diabetes, which indicate differences between women and men in mean differences (WM-βs) in amount of subcutaneous and visceral adipose tissue. Statistically significant differences between the sexes are typed in bold. *P value <0.05

Model 1: adjusted for age and height
Model 2: additionally adjusted for physical activity, healthy diet score, educational level, alcohol consumption and smoking status
Model 3: additionally adjusted for the use of medication known for weight gain and/or loss as possible side effect

For each potential confounder included, an interaction term (sex by potential confounder) was incorporated in the same model

ESM Figure 1 Overview of used techniques, locations, and implications

Technique	Location	Measures of body composition	Clinical implication of body composition measure
**DEXA**	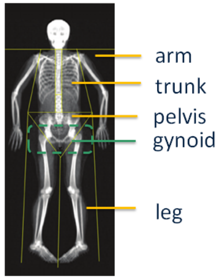	Total fat mass (g; all regions)<br>Trunk fat mass (g; trunk and pelvis)<br>Peripheral fat mass (g; arms and legs)<br>Gynoid fat mass (g)<br>Total lean mass (g; all regions)<br>Peripheral lean mass (g; arms and legs)	Total and trunk fat mass: metabolically disadvantageous (1,2)<br>Peripheral fat mass: less metabolically disadvantageous than total and trunk fat mass (3)<br>Gynoid fat mass: unclear (4,5)<br>Total and peripheral lean mass: unclear (6)
**MRI**	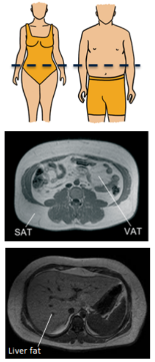	Subcutaneous adipose tissue (cm²; SAT)<br>Visceral adipose tissue (cm²; VAT)<br>Liver fat percentage (%)	SAT: unclear (7)<br>VAT: metabolically disadvantageous (3)<br>Liver fat: metabolically disadvantageous (1,3)
**Tape**	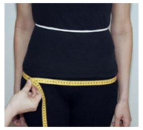	Hip circumference (cm)	Hip circumference: metabolically advantageous (8,9)

DEXA=dual-energy X-ray absorptiometry; MRI=magnetic resonance imaging. For DEXA-derived measures of body composition head fat or lean mass was not taken into account.

1) Sattar N, et al. BMC Med. 2014; 12: 123. 2) Santos LC, et al. Med J. 2008 Mar 6;126(2):82-6. 3) Power M, et al. Br J Nutr. 2008 May;99(5):931-40. 4) Wiklund P, et al. J Clin Endocrinol Metab. 2008 Nov;93(11):4360-

6. 5) FMcCarty M. Elsevier Medical Hypotheses 2003; 61(2): 173-176. 6) Rehunen S, et al. Diabetes Metab. 2021;47(6):101219. 7) Brand T, et al. J Clin Endocrinol Metab. 2021;106(10):e3881-e9. 8) Snijder MB et al. Am J Clin Nutr. 2003;77(5):1192-7. 9) Jayedi, A. et al. BMJ. 2022;376: e067516
